# Assessment of questionnaire-based PCB exposure focused on food frequency in birth cohorts in Japan

**DOI:** 10.1007/s11356-016-8119-6

**Published:** 2016-11-22

**Authors:** Akifumi Eguchi, Masae Otake, Masamichi Hanazato, Norimichi Suzuki, Yoshiharu Matsuno, Hiroko Nakaoka, Emiko Todaka, Chisato Mori

**Affiliations:** 1 Center for Preventive Medical Sciences, Chiba University, Inage-ku Yayoi-cho 1-33, Chiba City, Japan; 2 Department of Bioenvironmental Medicine, Graduate School of Medicine, Chiba University, Chuo-ku Inohana 1-8-1, Chiba City, Japan

**Keywords:** PCBs, Human serum, Food frequency, Diet

## Abstract

We investigated the relationship between food frequency questionnaire (FFQ) responses and serum polychlorinated biphenyl (PCB) levels of mothers and fathers recruited from the Chiba Regional Center, which is one of the 15 regional centers of the Japan Environment and Children’s Study (mothers: *n* = 1477, fathers: *n* = 219). The expected PCB values were estimated from the participants’ FFQ answers and medical records (age, body mass index and number of deliveries). Based on the stepwise forward selection results of Bayesian regression models, age and fish and egg consumption were positively associated with PCB concentrations and a number of deliveries were negatively associated with PCB concentrations in mothers, whereas only age was positively associated with PCB concentrations in fathers.

These findings indicated that the estimation of daily dietary intake may be useful for the prediction of PCB concentration for mothers.

## Introduction

The health effects of fetal and child environmental exposure to chemicals are a major concern, and several cohort studies have been conducted worldwide (Ayotte et al. 2003; Govarts et al. 2012; James et al. 2002). Polychlorinated biphenyls (PCBs) are known to affect the endocrine systems in humans. Despite being banned in 2001 by the Stockholm Convention (UNEP 2001), PCBs widely persist in wildlife and humans because of their lipophilic properties, low water solubility, and bioaccumulation in fatty tissues (Safe 1994). PCBs are reported to have numerous adverse human health effects, including reproductive (Leoni et al. 1989), developmental (Jorissen 2007), immunological (Glynn et al. 2008), and neurological (Prince et al. 2006). Food is a major source of exposure to PCBs and dioxins, which are related compounds (Caspersen et al. 2013; Kvalem et al. 2009). It has been reported that the consumption of fish was positively associated with serum levels of total PCBs among Japanese women (Tsukino et al. 2006), suggesting that human PCB contamination levels are strongly affected by eating habits.

Self-administered, semi-quantitative food frequency questionnaire (FFQ) has been used to assess the nutrient and food intake of subjects in large-scale epidemiologic studies from several countries, including Japan (Sasaki et al. 2003; Tsugane et al. 2003a; Willett 1994). If groups at high risk for PCB contamination could be identified by an FFQ approach, without chemical analyses, significant cost and time could be saved. Various prediction models for PCB concentrations in humans have been generated using dietary intake (Kvalem et al. 2009; Laden et al. 1999; Xue et al. 2014); however, few studies have addressed this issue in Japanese populations.

The Japan Environment and Children’s Study (JECS) is a nationwide birth cohort study of 100,000 parent–child pairs that evaluates the impact of various environmental factors on children’s health and development (Kawamoto et al. 2014). Here, in an adjunct study of JECS, we evaluated the relationship between FFQ responses and serum PCB contamination levels of participants at the Chiba Regional Center.

## Materials and methods

This study was approved by the Ethics Committee of Chiba University, Japan. Data of participants (age, body mass index [BMI], and a number of deliveries) and FFQ were completed by mothers (*n* = 1716) and fathers (*n* = 318) recruited from the Chiba Regional Center, which is one of the 15 regional centers of JECS. Age, BMI, and reproductive information were collected from medical record information. Participants who had completed <60% of the FFQ, outlier of energy intake (below 600 kcal/day or above 4000 kcal/day), and not completed the medical record information (age, BMI, and reproductive information) were excluded from the analysis. Finally, the remaining 1477 mothers (86.1%) and 219 fathers (68.9%) were used for all analysis (Table 1).

**Table 1:** Subjects’ characteristics, FFQ data in fathers and mothers recruited in Chiba Regional Center of JECS

	Mothers (*n* = 1477)							Fathers (*n* = 219)						
	Mean	Standard deviation	0%	25%	50%	75%	100%	Mean	Standard deviation	0%	25%	50%	75%	100%
Age	31.0	5.0	17.0	28.0	31.0	35.0	46.0	32.9	6.0	20.0	29.0	32.0	37.0	64.0
BMI	21.4	3.2	14.6	19.2	20.7	22.7	39.9	24.4	3.9	16.9	21.9	23.8	26.2	39.0
Height	158.2	5.4	140.0	155.0	158.0	162.0	178.0	171.4	5.7	158.0	167.3	171.5	175.0	190.0
Weight	53.5	8.9	37.0	48.0	52.0	57.0	106.0	71.8	12.3	47.3	63.0	70.0	78.7	110.0
A number of deliveries	0.9	0.8	0.0	0.0	1.0	1.0	5.0							
Energy (kcal)	1845	583	685	1426	1758	2153	3973	2208	625	1056	1756	2158	2593	3920
Intakes of foods (g/day/kcal)														
Eggs	15.4	13.1	0.0	7.6	13.3	19.6	194.3	15.7	20.4	0.0	5.8	10.7	18.3	145.9
Fats and oils	6.0	2.4	1.2	4.3	5.6	7.2	26.3	5.4	3.9	0.0	3.0	4.5	6.9	36.5
Fish and shellfish	22.1	15.0	0.0	12.4	20.0	28.8	154.8	22.0	18.5	0.0	8.0	17.6	30.8	119.8
Fruits	89.1	70.5	0.0	39.6	74.8	121.7	740.2	53.2	71.4	0.0	10.3	33.3	72.3	548.1
Meats	38.9	19.5	0.0	25.2	36.1	49.0	143.9	48.6	36.8	0.0	23.9	39.7	60.2	241.0
Milks	138.8	126.2	0.0	59.6	108.6	171.2	914.5	94.3	163.2	0.0	16.4	45.4	96.7	1315
Potatoes and starches	13.9	9.5	0.0	7.3	12.1	18.4	89.4	11.0	11.0	0.0	3.6	8.4	14.7	88.5
Vegetables	106.0	62.7	0.0	64.1	91.4	133.8	829.0	82.1	78.5	0.0	34.4	57.2	110.4	492.9
Water	217.7	313.9	0.0	22.1	97.1	286.0	2386	174.5	322.7	0.0	22.3	68.8	215.4	3585

The FFQ used in the JECS is a validated, self-administered diet questionnaire that has been evaluated in previous studies with respect to nutrient factors (Ishihara et al. 2003; Iso et al. 2003; Tsubono et al. 2003; Tsugane et al. 2003b). The FFQ was a self-administered questionnaire comprising 13 pages. The FFQ included food lists (cereals, eggs, fat and oils, fish and shellfish, fruits, meats, mushrooms, milks, potatoes and starches, and vegetables), standard units (volumes; 0.5 for small, 1.0 for medium, and 1.5 for large), portion sizes (e.g., 30 g portion^−1^), and frequencies (ranging from less than once per month to more than seven times per day). Moreover, eating habits (e.g., frequency of having breakfast, eating out, and speed of eating) were assessed by the questionnaire. The subjects reported their intake frequency and portions of consumption for the past year (Sakurai et al. 2004; Takasuga et al. 2004). Consumption amounts were calculated using the frequencies, standard units, and portion sizes for the various food items of the FFQ. This amount was then energy adjusted using the energy density method (Drewnowski and Specter 2004).

During the mother’s pregnancy, blood samples of 30 mL were drawn once from both mothers and fathers. PCB levels were analyzed according to the procedures previously reported (Jotaki et al. 2011; Mori et al. 2014). Approximately 1 g of serum was hydrolyzed with 1 M potassium hydroxide that was diluted in ethanol for 18 h and extracted three times with hexane. The extract was washed three times with distilled water and then dehydrated with anhydrous sodium sulfate. The solution was concentrated by evaporation to approximately 2 mL, eluted through a Florisil column (Florisil PR; GL Sciences, Tokyo, Japan) with 50 mL hexane, evaporated to a final volume of 1 mL, and analyzed by packed-column gas chromatography with an electron capture detector (GC/ECD; GC-17A, Shimadzu, Kyoto, Japan). The PCB concentration was calculated as the sum of the major eight peaks shown after *p*,*p′*-DDE on the chromatogram, based on the peaks detected in a Kanechlor mixture standard (Kanechlor 300, 400, 500, and 600 = 1:1:1:1) (JIS K 0093: 2006; Mori et al. 2014). The detection limit was 0.1 ng g^−1^ wet weight; samples below the lower level of detection were assigned to a value of 0.05 ng g^−1^ (JIS K 0093: 2006). The total PCB concentrations that were analyzed by packed GC/ECD were strongly correlated (*R* ^2^ > 0.996) with values analyzed by GC-HRMS in our previous study (Mori et al. 2014).

### Statistical analysis

The relationships between body mass index (BMI), age, FFQ, and PCB concentrations (wet weight) were analyzed by the Bayesian linear regression modeling separately by gender for both parents. The PCB concentration data was assessed for normal distribution with the Shapiro–Wilkes test and was not normally or log-normally distributed but was right-skewed and showed slightly heavy tails. Due to the distribution of PCB concentration, Bayesian linear regression modeling was conducted using a No-U-Turn Sampler variant of the Hamiltonian Markov Chain Monte Carlo (Hoffman and Gelman 2014) approach that was implemented in Stan version 2.8.0 and accessed through the rstan packing (Stan Development Team 2015) of the R program (R Core Team 2014). This approach was chosen because Bayesian linear regression modeling is sufficiently tolerant to handle skewed and heavy-tailed distributions. We used the non-informative *N* (0, 100^2^) prior distribution as the prior mean PCB concentration in all models.

In the Bayesian linear regression modeling, we ran three chains from random initializations each with 10,000 samples, thinned to every 2 samples, and discarded the first 5000 samples from each as burn-in. We checked convergence by ensuring that R-hat, an estimate of the potential scale reduction of the posterior if sampling were to be infinitely continued, was below 1.1 (Gelman et al. 2014). The reported empirical 95% credible intervals (95% CI) represent the 2.5th to 97.5th percentiles of the highest posterior density interval calculated from the posterior samples.

Following this, the predictive accuracy from various models can be compared using the Watanabe–Akaike information criterion (WAIC) (Vehtari and Gelman 2014; Watanabe 2010):

$$ \mathrm{WAIC}=-2\left(\widehat{lpd}-{\widehat{P}}_{\mathrm{waic}}\right) $$

where $\widehat{lpd}$ represents the computed log pointwise predictive density and ${\widehat{P}}_{\mathrm{waic}}$ is the estimated effective number of parameters (Vehtari and Gelman 2014; Watanabe 2010). The model with the lowest WAIC is selected to achieve a trade-off between model complexities. WAIC was derived based on a singular learning theory as an asymptotically unbiased approximation to the out-of-sample prediction error and is a generalization of Akaike’s information criterion that is applicable to both regular and singular statistical models (Watanabe 2010). The variable selection in the Bayesian linear regression modeling was evaluated based on the stepwise forward selection method by WAIC.

## Results and discussion

The mean ± standard deviation (SD) serum concentrations of PCBs in fathers and mothers were 0.38 ± 0.21 and 0.28 ± 0.16 ng g^−1^ wet weights, respectively (Table 2). The mean concentrations of PCBs stratified by a number of deliveries in mothers are shown in Table 2. PCB concentrations in the fathers were higher than in the mothers, and among the mothers, serum PCB concentrations were lower as the number of deliveries was greater. PCB levels in this study are comparable or slightly lower than the serum levels of previous cohort studies in Japan, USA, and European countries (Table 3) (Govarts et al. 2012; Inoue et al. 2006; Patterson et al. 2009). Based on the stepwise forward selection results, consumption of fish and eggs was selected along with age and number of delivery for the model of mothers by WAIC (Tables 4 and 6). In mothers, age [*β* = 0.26, 95% CI (0.23, 0.29)] and fish [*β* = 0.055, 95% CI (0.029, 0.082)] and egg consumptions [*β* = 0.025, 95% CI (−0.00027, 0.052)] were positively and a number of deliveries [*β* = −0.18, 95% CI (−0.21, −0.16)] was negatively associated with PCB concentrations in mothers (Table 6). However, in our analysis, only age [*β* = 0.25, 95% CI (0.19, 0.32)] was selected for the fathers’ model (Tables 5 and 6).

**Table 2:** PCB concentrations (ng/g wet wt.) in serum from fathers and mothers

	Mean	Standard deviation	Min	25%	50%	75%	Max
Father (*n* = 219)	0.38	0.21	0.05	0.23	0.32	0.49	1.2
Mother (*n* = 1477)	0.28	0.16	0.05	0.18	0.25	0.35	1.3
Delivery status of mothers							
A number of deliveries: 0 (*n* = 550)	0.32	0.17	0.05	0.21	0.28	0.39	1.3
A number of deliveries: 1 (*n* = 644)	0.26	0.14	0.05	0.17	0.24	0.33	1.3
A number of deliveries: 2 (*n* = 234)	0.23	0.14	0.05	0.14	0.21	0.29	0.79
A number of deliveries: 3 (*n* = 37)	0.22	0.15	0.05	0.12	0.19	0.25	0.71
A number of deliveries: 4 (*n* = 10)	0.15	0.082	0.05	0.068	0.16	0.22	0.26
A number of deliveries: 5 (*n* = 2)	<0.1

**Table 3:** Comparison of serum concentrations of PCBs (pg g^−1^ wet wt.) in participants analyzed in this study with previous data

	Age	Mean	Standard deviation	Min	25%	50%	75%	Max	References
Father (*n* = 219)	32.9 ± 6.0	0.38	0.21	0.05	0.23	0.32	0.49	1.2	This study
Mother (*n* = 1477)	31.0 ± 5.0	0.28	0.16	0.05	0.18	0.25	0.35	1.3	This study
Japanese mother (*n* = 89)*	20–29 years (50.6%) 30–39 years (44.9%) 40–49 years (5.5%)	0.36		0.062	0.21	0.30	0.45	1.3	Inoue et al. 2006
Mothers from 12 European birth cohorts (*n* = 7762: CB153)	>20% of mothers were <25 years of age at delivery and in two populations >20% were >35 years of age	0.18	0.16			0.14			Govarts et al. 2012
National Health and Nutrition Examination Survey (2003–2004): USA	12–19 years (31.2%) 20–39 years (24.5%) 40–59 years (20.3%) +60 years (24.0%)								
Male (*n* = 928)		0.835**				0.814	1.62		Patterson et al. 2009
Female (*n* = 938)		0.805**				0.824	1.65		Patterson et al. 2009

*Adjusted by reported average serum lipid level (7.9 g/L) (Longnecker et al. 2003)

**Geometric mean

**Table 4:** Selection of prediction model based on mothers’ dataset by WAIC

	WAIC
PCB ~ age	−1843.5
PCB ~ age + a number of deliveries	−2012.9
PCB ~ age + a number of deliveries + BMI	−2011.4
PCB ~ age + a number of deliveries + cereals	−2012.2
PCB ~ age + a number of deliveries + potatoes and starches	−2011.5
PCB ~ age + a number of deliveries + vegetables	−2013.3
PCB ~ age + a number of deliveries + fruits	−2011.4
PCB ~ age + a number of deliveries + fish and shellfish	−2028.5
PCB ~ age + a number of deliveries + meats	−2011.3
PCB ~ age + a number of deliveries + eggs	−2014.2
PCB ~ age + a number of deliveries + milk	−2011.6
PCB ~ age + a number of deliveries + fats and oils	−2011.5
PCB ~ age + a number of deliveries + fish and shellfish + BMI	−2027.5
PCB ~ age + a number of deliveries + fish and shellfish + cereals	−2027.0
PCB ~ age + a number of deliveries + fish and shellfish + potatoes and starches	−2027.8
PCB ~ age + a number of deliveries + fish and shellfish + vegetables	−2027.9
PCB ~ age + a number of deliveries + fish and shellfish + fruits	−2027.0
PCB ~ age + a number of deliveries + fish and shellfish + meats	−2026.4
*PCB ~ age + a number of deliveries + fish and shellfish + eggs*	−*2030.2*
PCB ~ age + a number of deliveries + fish and shellfish + milk	−2028.3
PCB ~ age + a number of deliveries + fish and shellfish + fats and oils	−2026.7
PCB ~ age + a number of deliveries + fish and shellfish + eggs + BMI	−2029.2
PCB ~ age + a number of deliveries + fish and shellfish + eggs + cereals	−2028.4
PCB ~ age + a number of deliveries + fish and shellfish + eggs + potatoes and starches	−2029.7
PCB ~ age + a number of deliveries + fish and shellfish + eggs + vegetables	−2029.4
PCB ~ age + a number of deliveries + fish and shellfish + eggs + fruits	−2029.1
PCB ~ age + a number of deliveries + fish and shellfish + eggs + meats	−2028.3
PCB ~ age + a number of deliveries + fish and shellfish + eggs + milk	−2030.0
PCB ~ age + a number of deliveries + fish and shellfish + eggs + fats and oils	−2028.4

*Italicized model was selected with the smallest WAIC

**Table 5:** Selection of prediction model based on fathers’ dataset by WAIC

	WAIC
*PCB ~ age*	*−172.8*
PCB ~ age + BMI	−172.4
PCB ~ age + cereals	−171.0
PCB ~ age + potatoes and starches	−172.5
PCB ~ age + vegetables	−169.9
PCB ~ age + fruits	−171.9
PCB ~ age + fish and shellfish	−172.0
PCB ~ age + meats	−170.9
PCB ~ age + eggs	−171.4
PCB ~ age + milks	−171.7
PCB ~ age + fats and oils	−170.7
PCB ~ age + potatoes and starches + BMI	−172.2
PCB ~ age + potatoes and starches + cereals	−171.2
PCB ~ age + potatoes and starches + vegetables	−170.0
PCB ~ age + potatoes and starches + fruits	−171.0
PCB ~ age + potatoes and starches + meats	−171.1
PCB ~ age + potatoes and starches + fish and shellfish	−171.0
PCB ~ age + potatoes and starches + eggs	−171.3
PCB ~ age + potatoes and starches + milk	−171.4
PCB ~ age + potatoes and starches + fats and oils	−171.2

*Italicized model was selected with the smallest WAIC

**Table 6:** Empirical mean (mean), standard deviation of the mean (SD), and quantiles for each variable in selected prediction model for mothers and fathers

	Mothers				Fathers			
	Coefficient	Standard deviation	95% lower	95% upper	Coefficient	Standard deviation	95% lower	95% upper
Intercept	−1.32	0.013	−1.34	−1.29	−1.0	0.032	−1.1	−0.93
Age	0.26	0.014	0.23	0.29	0.25	0.033	0.19	0.32
A number of deliveries	−0.18	0.013	−0.21	−0.16	NA			
Fish and shellfish	0.055	0.013	0.029	0.082	Omitted			
Eggs	0.025	0.013	−0.00027	0.052	Omitted			

“Omitted” indicates factors eliminated by stepwise procedures

*NA* not analyzed

The relationship between PCB concentration and age is commonly assumed to result from the continued exposure to or intake of PCB (Mori et al. 2014; Wolff et al. 2007) and the long half-lives of PCBs in the human body (Ritter et al. 2011). In a former study, our group also reported that delivery experience and lactation affected the PCB concentration level in adults (Mori et al. 2014), indicating women may excrete PCBs through delivery and lactation (Agudo et al. 2009; Kang et al. 1997).

Studies have demonstrated that the dietary intake of fish is a major pathway for human exposure to PCBs (Caspersen et al. 2013; Kvalem et al. 2009; Sjodin et al. 2000). Fitzgerald et al. (2004) reported that Mohawk women had high serum levels of PCBs that were associated with consumption of local fish and not with the exposure to other sources, like residential soil, lived sites, and the other foodstuff (Fitzgerald et al. 2004). Furthermore, it was reported that fish and shellfish accounted for 30–84% of the dioxin toxic equivalences from food intake by a Japanese total-diet study (Sasamoto et al. 2006), indicating that dioxin and dioxin-like PCB intake also occurs through fish and shellfish. Sasamoto et al. (2006) reported that meat and egg consumption comprised the second highest contribution to dietary intake of dioxins and dioxin-like PCBs, indicating that egg consumption may be associated with higher serum dioxin and dioxin-like PCB concentrations. The Bureau of Social Welfare and Public Health, Tokyo Metropolitan Government, showed the levels of PCBs in meat and eggs were below the detection limit in a food contamination survey (Bureau of Social Welfare and Public Health, Tokyo Metropolitan Government, 2015); however, continuous monitoring of PCBs in these foodstuffs might be needed.

In mother’s models, effect sizes of food intakes were 1 order lower than that of age and delivery experience, indicating the effect of food intake on serum PCB concentrations was weaker than that of age and number of deliveries. However, fish intake was included in the final model in mothers, indicating FFQ might be useful to estimate the PCB levels. In the father’s model, only age is predictive of serum PCB concentrations. Due to lack of information about smoking habits and limitation of sample size of father, development of improved predictive models and detailed investigations are required in future study.

Limitations of this study were lack of data about breast-feeding and smoking habit. Several studies have reported that breast-feeding is one of the major elimination pathways of persistent organic pollutants (POPs) (Abraham et al. 1996; Milbrath et al. 2009; Schecter et al. 1996), and after 6 months of breast-feeding, approximately 20% or more of the maternal body burden of these POPs may be transferred to children from mothers (Landrigan et al. 2002; Milbrath et al. 2009; Niessen et al. 1984). These results indicated that the number of deliveries is most likely a surrogate for breast-feeding. It was also reported that the active smoking was negatively affected to levels of PCB and dioxin in serum (Chen et al. 2005; Milbrath et al. 2009) through increased induction of dioxin-degrading enzymes, like cytochrome P450, by activation of the aryl hydrocarbon receptor by polycyclic aromatic hydrocarbons in tobacco smoke (Milbrath et al. 2009; Zevin and Benowitz 1999). Thus, information about smoking status and breast-feeding will be needed to develop more accurate predictive models in any future study.

## Conclusion

In the current study, we developed a preliminary model to estimate the serum PCB levels from FFQ and other demographic/reproductive data collected from medical records. Finally, in mothers, age and consumption of fish and eggs were positively associated and the number of deliveries was negatively associated with PCB concentration. On the other hand, only age was positively associated with PCBs in fathers in this study.

In previous studies, it was shown that age was strongly related to exposure of PCBs, due to intake of PCB and long half-lives of PCBs in the human body. On the other hand, only women might excrete the PCBs through delivery and lactation. Dietary exposure to PCBs, especially in relation to fish consumption, was also reported, indicating out results that supported the previous cohort studies, even in the current Japanese cohort study. While the limitations about the smoking habits and breast-feeding existed, this study shows that the FFQ may help predict PCB levels in serum from women. In the future, development of the more accurate predictive models using information about the smoking status and breast-feeding was needed.
